# Initiation of a Ring Approach to Infection Prevention and Control at Non-Ebola Health Care Facilities — Liberia, January–February 2015

**Published:** 2015-05-15

**Authors:** Tolbert Nyenswah, Moses Massaquoi, Miatta Zenabu Gbanya, Mosoka Fallah, Fred Amegashie, Adolphus Kenta, Kumblytee L. Johnson, Disu Yahya, Mehboob Badini, Lacina Soro, Carmem L. Pessoa-Silva, Isabelle Roger, Linda Selvey, Kristin VanderEnde, Matthew Murphy, Laura A. Cooley, Sonja J. Olsen, Athalia Christie, John Vertefeuille, Thomas Navin, Peter McElroy, Benjamin J. Park, Eric Esswein, Ryan Fagan, Frank Mahoney

**Affiliations:** 1 Ministry of Health and Social Work, Liberia; 2 Montserrado Incident Management System; 3 Montserrado County Health Team; 4 C.H. Rennie Hospital; 5 African Union; 6 World Health Organization; 7 CDC; 8 Epidemic Intelligence Service; 9 National Institute for Occupational Safety and Health, CDC; 10 University of Witwatersrand

From mid-January to mid-February 2015, all confirmed Ebola virus disease (Ebola) cases that occurred in Liberia were epidemiologically linked to a single index patient from the St. Paul Bridge area of Montserrado County (1). Of the 22 confirmed patients in this cluster, eight (36%) sought and received care from at least one of 10 non-Ebola health care facilities (HCFs), including clinics and hospitals in Montserrado and Margibi counties, before admission to an Ebola treatment unit. After recognition that three patients in this emerging cluster had received care from a non-Ebola treatment unit, and in response to the risk for Ebola transmission in non-Ebola treatment unit health care settings (2), a focused infection prevention and control (IPC) rapid response effort for the immediate area was developed to target facilities at increased risk for exposure to a person with Ebola (Ring IPC). The Ring IPC approach, which provided rapid, intensive, and short-term IPC support to HCFs in areas of active Ebola transmission, was an addition to Liberia’s proposed longer term national IPC strategy, which focused on providing a comprehensive package of IPC training and support to all HCFs in the country. This report describes possible health care worker exposures to the cluster’s eight patients who sought care from an HCF and implementation of the Ring IPC approach. On May 9, 2015, the World Health Organization (WHO) declared the end of the Ebola outbreak in Liberia.*

## St. Paul Bridge Cluster

The eight Ebola patients who sought care from an HCF ranged in age from 10 to 56 years; three were female. Only two of the eight were on a contact tracing list of persons with known prior Ebola exposure when they went to the HCF. Two patients died in the community and were never admitted to an Ebola treatment unit. For the other six, a median of 1 day passed (range = 0–9 days) between the first visit to an HCF and their admission to an Ebola treatment unit. For three patients with available data, a fever (defined as a temperature >100.4°F [>38°C] taken with an infrared thermometer) was not recorded on arrival at the HCF. Of the eight patients, seven subsequently died from Ebola.

Overall, 166 non-Ebola treatment unit health care workers in 10 HCFs possibly were exposed to the eight Ebola patients (Figure). The nature of their possible exposures varied, including providing treatment or performing laboratory tests on blood specimens of Ebola patients, or cleaning a room or mattress where an Ebola patient had slept. All 166 were placed on contact lists. Of these, 86 (52%) were placed under home-based quarantine or precautionary observation at an HCF because of relatively higher risk exposures, while the remainder were monitored by contact tracers, either at home or at their place of employment (Figure). One clinic was closed entirely (clinic D) (Figure) during the postexposure period of observation, and one inpatient section of a hospital was closed (hospital D) (Figure). One health care worker developed Ebola after debriding and suturing the lacerations of an Ebola patient who had sought care for treatment of injuries sustained in a fight, and both the patient and the health care worker died. None of the other 165 health care workers developed Ebola.

Observations of the 10 HCFs found that health care worker exposures could have occurred for multiple reasons. Triage systems were inadequate with limited or no triage or isolation structures, no use or inappropriate use of infrared thermometers, limited ability to elicit accurate contact exposure history, and an incomplete understanding of the case definition (suspected or probable) by some staff members. Additional challenges included no use or inappropriate use of personal protective equipment, insufficient staff to support IPC activities, and inconsistent oversight by IPC partners.

## Ring IPC Strategy

Ring IPC was a collaboration of the Liberian Ministry of Health National and Montserrado Incident Management Systems, Montserrado and Margibi county health teams, CDC, WHO, the African Union, and nongovernmental organization partners under the Liberia IPC Task Force. On January 30, members of the IPC Task Force met to formalize components of the Ring IPC approach, including identification of target HCFs, a focus on triage, involvement of external staff members to support triage, and coordination and definition of roles among partners. The purpose of Ring IPC was to provide intensive IPC support (3,4) to HCFs in areas of active Ebola transmission, thus forming a strategically placed protective ring of intensified IPC attention around persons with known Ebola to help break the chain of transmission. This strategy entailed selecting target HCFs for Ring IPC intervention based on known health care worker exposure to an Ebola patient, neighboring HCFs around the HCF that treated a patient, or HCFs in close proximity to the residence of a patient with confirmed Ebola. Next, rapid IPC needs assessments were conducted at these HCFs using Ministry of Health and Social Work (MOHSW) approved assessment tools (5). These assessments focused on triage procedures and personal protective equipment use, and found inadequate or absent triage and isolation structures, gaps in the personal protective equipment supply chain, and a need for general IPC training in addition to specialized triage training.

What is already known on this topic?

The adoption of essential infection prevention and control (IPC) practices among health care workers, such as hand washing and proper use of personal protective equipment, is crucial to interrupting the transmission of Ebola.

What is added by this report?

From mid-January to mid-February 2015, all confirmed Ebola cases in Liberia were epidemiologically linked to a single index patient. Of the 22 confirmed patients in this cluster, eight (36%) sought and received care from at least one of 10 non-Ebola health care facilities. During this time, a focused IPC response effort, termed Ring IPC, was developed collaboratively to target health care facilities at increased risk for exposure to a person with Ebola. Rapid, intensive, and short-term IPC support was provided at these health care facilities following rapid needs assessments focused on triage procedures and personal protective equipment use.

What are the implications for public health practice?

The implementation of Ring IPC in Liberia might offer a useful model for rapid response to Ebola virus transmission and health care worker exposure in other settings. A comprehensive strategy remains critical to raising the level of IPC capacity nationwide; however, an appropriately targeted Ring IPC approach might be an effective supplemental strategy to focus IPC support in response to clusters of disease.

## Training and Equipment

Identified challenges were addressed by the national IPC Task Force developing training that targeted key personnel. Triage training, based on existing MOHSW-approved IPC training materials, was developed and provided to 47 African Union clinicians. These clinicians were deployed to 36 target HCFs in Monteserrado County to provide onsite daily triage mentoring and support for the duration of the high-risk exposure monitoring period, or for at least 2 weeks. Three nurses, previously employed by an Ebola treatment unit, provided similar triage support for one hospital. In addition, three 1-day triage training sessions were provided for more than 125 staff members working in three target HCFs. In Margibi County, a 1-day triage training session was conducted for 11 staff members working in five target HCFs, and African Union staff and nurses or other county health staff members provided ongoing triage mentoring and IPC support to seven target HCFs. This intensive IPC approach served to alert health care workers to recent Ebola virus transmission in their communities, identify additional contacts at HCFs where Ebola virus exposure had occurred, and provide a secondary source (in addition to contact tracing) of information on the health status of exposed health care workers.

In response to heightened awareness of clinic needs, partners provided personal protective equipment and other essential IPC supplies to target facilities. Ring IPC partners in Montserrado County and the national IPC Task Force initiated an emergency release of a 1-month supply of personal protective equipment to priority clinics. Nongovernmental organization partners assessed and constructed triage structures when needed.

## Initiation of Rings

During January 23–February 9, in response to the ongoing St. Paul Bridge cluster, four IPC rings were initiated in Liberia, three in Montserrado County and one in Margibi County (Figure). The first ring was initiated 4 days after recognition that a facility had provided care to an Ebola patient; subsequent rings were initiated within 2 days after recognition of other Ebola patients. In total, 59 target HCFs were identified, 52 in Montserrado County (out of a total of 294 HCFs) and seven (out of a total of 32) in Margibi County. There was an average of 15 HCFs per ring (range = 3–31).

Overall, Ring IPC efforts appeared to be associated with an increase in the identification and isolation of suspected or probable Ebola patients. For example, three probable Ebola patients were identified through triage during training conducted at one target HCF in Montserrado County. Only one of the 166 exposed health care workers in the St. Paul Bridge cluster became infected with Ebola. This low prevalence of secondary infection among health care workers suggests that basic infection prevention principles were being observed by health care workers during this period. Nevertheless, triage was not always completely successful; the one health care worker who became infected with Ebola after Ring IPC activities were initiated actually sought care at his place of employment, an identified target HCF, and was permitted to enter without first being properly triaged as a probable or suspect Ebola patient.

### Discussion

Included among the Ebola response efforts in Liberia was the creation in early September 2014 of a national IPC Task Force to support the MOHSW. The IPC Task Force served as a coordinating body to facilitate IPC planning and implementation of activities in both health care and non–health care facilities, as well as providing IPC guidance and technical assistance through policy development and standardization of IPC training and implementation tools consistent with MOHSW priorities. The national IPC strategy had focused on providing a comprehensive package of IPC training and support, through trained IPC specialists, at major health facilities throughout the country because of widespread Ebola transmission occurring at the time. This strategy includes promoting essential IPC practices among health care workers, such as hand washing and proper use of personal protective equipment. Although a comprehensive strategy remains critical to raising the level of IPC capacity nationwide, an appropriately targeted Ring IPC approach might be an effective supplemental strategy to focus IPC support in response to clusters of disease.

The public health intervention described in this report was rapidly implemented and integrated into Liberia’s national Ebola response as a result of coordinated, collaborative efforts by multiple partners. Coordination and collaboration among the national Incident Management System, county health teams, CDC, WHO, African Union and nongovernmental organization partners was key to identifying gaps in IPC needs and preventing duplication of efforts. The initial ring was coordinated by the IPC Task Force under MOHSW leadership. In subsequent rings, the national Incident Management System and county health departments joined efforts with CDC, WHO, African Union, and multiple nongovernmental organization partners participating in initial discussions, planning, and rapid IPC assessments. In general, HCFs welcomed additional training, personal protective equipment provision, and triage mentoring and support. The placement of IPC staff members trained in triage at target HCFs following training was readily adopted by clinic staff.

The implementation of Ring IPC in Liberia might offer a useful model for rapid response to Ebola virus transmission and health care worker exposure in other settings. This approach, however, might be most appropriate at the beginning or near the end of an outbreak, when specific chains of transmission can be identified and when HCFs can be identified and targeted based on their risk for encountering an Ebola patient when there is known active transmission in their geographical area. Urban settings present challenges to this approach, because persons might seek care at HCFs outside of their immediate community. Although limitations in both supplies (personal protective equipment and infrared thermometers) and human resources (appropriately trained personnel) might inhibit a timely response to initiating IPC activities, the Ring IPC approach might be used to prioritize these limited resources.

The Ring IPC approach was developed rapidly and collaboratively in response to an urgent public health need; as such, data were not collected and aggregated systematically across all facilities, potentially limiting the generalizability of these results. Nonetheless, as a result of Ring IPC efforts, health care workers at HCFs in areas with recent active transmission are now better equipped and trained to rapidly triage, isolate, and refer suspected and probable Ebola patients to appropriate Ebola treatment unit facilities. As Liberia looks ahead, a new culture of IPC can be incorporated into the health system; a Ring IPC approach might be useful in minimizing the transmission in non-Ebola HCFs should new cases of Ebola occur.

## Figures and Tables

**FIGURE:**
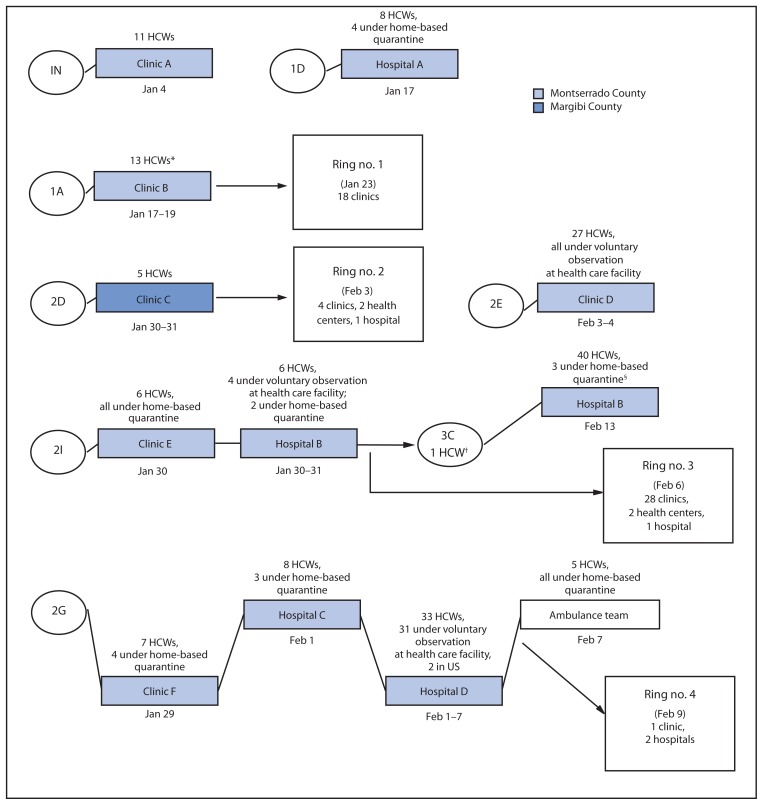
Health care workers (HCWs) with exposure to eight Ebola patients at non-Ebola treatment units and targeted infection prevention and control (Ring IPC) initiation — Montserrado and Margibi counties, Liberia, January–February 2015 **Abbreviations:** IN = index patient for St. Paul Bridge cluster; 1A and 1D = other patients identified as first generation of cluster; 2D, 2E, 2G, and 2I = patients identified as second generation; 3C = patients identified as third generation. * A sister of patient 1A who worked at Clinic B was exposed in her role as family caregiver. ^†^ 3C, one of the 2 HCWs under quarantine at home, sought care at his place of employment while symptomatic. ^§^ The home-based quarantine period was extended for 3 patients previously under home-based quarantine from exposure to 3C.

## Footnotes

* Additional information available at http://www.who.int/mediacentre/news/statements/2015/liberia-ends-ebola/en/.
